# Postnatal High-Fat Diet Increases Liver Steatosis and Apoptosis Threatened by Prenatal Dexamethasone through the Oxidative Effect

**DOI:** 10.3390/ijms17030369

**Published:** 2016-03-11

**Authors:** Ying-Hsien Huang, Chih-Jen Chen, Kuo-Shu Tang, Jiunn-Ming Sheen, Mao-Meng Tiao, You-Lin Tain, Chih-Cheng Chen, En-Wei Chu, Shih-Wen Li, Hong-Ren Yu, Li-Tung Huang

**Affiliations:** Department of Pediatrics, College of Medicine, Kaohsiung Chang Gung Memorial Hospital and Chang Gung University, No. 123, Ta-Pei road, Niaohsung, Kaohsiung 833, Taiwan; yhhuang123@yahoo.com.tw (Y.-H.H.); superarthy2@yahoo.com (C.-J.C.); tangl004@cgmh.org.tw (K.-S.T.); ray.sheen@gmail.com (J.-M.S.); tainyl@adm.cgmh.com.tw (Y.-L.T.); charllysc@cgmh.org.tw (C.-C.C.); 3823212123456789@yahoo.com.tw (E.-W.C.); violet7053@gmail.com (S.-W.L.); yuu2004taiwan@yahoo.com.tw (H.-R.Y.); litung.huang@gmail.com (L.-T.H.)

**Keywords:** high-fat diet, liver steatosis, apoptosis, dexamethasone

## Abstract

The objective of this study was to investigate cellular apoptosis in prenatal glucocorticoid overexposure and a postnatal high fat diet in rats. Pregnant Sprague-Dawley rats at gestational days 14 to 21 were administered saline (vehicle) or dexamethasone and weaned onto either a normal fat diet or a high fat diet for 180 days; in total four experimental groups were designated, *i.e.*, vehicle treated group (VEH), dexamethasone treated group (DEX), vehicle treated plus high-fat diet (VHF), and dexamethasone treated plus high-fat diet (DHF). Chronic effects of prenatal liver programming were assessed at postnatal day 180. The apoptotic pathways involved proteins were analyzed by Western blotting for their expressions. Apoptosis and liver steatosis were also examined by histology. We found that liver steatosis and apoptosis were increased in the DHF, DEX, and VHF treated groups, and that the DHF treated group was increased at higher levels than the DEX and VHF treated groups. The expression of leptin was decreased more in the DHF treated group than in the DEX and VHF treated groups. Decreased peroxisome proliferator-activated receptor-gamma coactivator 1α, phosphoinositide-3-kinase, manganese superoxide dismutase and increased malondialdehyde expression levels were seen in DHF treated group relative to the DEX treated group. The DHF treated group exhibited higher levels of oxidative stress, apoptosis and liver steatosis than the DEX treated group. These results indicate that the environment of high-fat diet plays an important role in the development of liver injury after prenatal stress.

## 1. Introduction

Maternally administered glucocorticoid results in changes in certain tissues (e.g., liver) that persist throughout life [1,2]. Prenatal dexamethasone programs expression of adipose tissue and increase hepatic lipid accumulation in liver on a high-fat diet [3,4]. It is also reported that the deleterious effects of high-fat diet on perinatal and post-weaning periods will be transmitted to adult rat offspring [4,5]. This leads to intrahepatic lipid accumulation and a decreased metabolic flexibility [6]. The more important issue is that prenatal dexamethasone and postnatal high fat diet synergistically induce programmed disease in adult offspring [7].

High-fat diet feeding can induce inflammation and oxidative stress [8]. The detrimental effect of high-fat diet in childhood or adult was fatty liver; tied to mitochondria of oxidative phosphorylation [9]. Peroxisome proliferator-activated receptor-gamma coactivator 1α (PGC-1 α) is an essential cofactor in mitochondrial biogenesis driving metabolic rate [10]. Induction of manganese superoxide dismutase (MnSOD), an antioxidant protein, was possibly stimulated via PGC-1 α, and this may be associated with lower proinflammatory cytokine activation, such as tumor necrosis factor α (TNFα) [11]. However, the association of oxidative stress, prenatal dexamethasone (DEX) and postnatal high-fat diet were unknown.

It was reported that there was no higher hepatocyte apoptosis in high-fat diet induced liver steatosis [12]. Another study showed that liver steatosis and oxidative stress resulted in hepatocyte apoptosis [13]. The association of hepatocyte apoptosis with prenatal DEX and postnatal high-fat diet was unknown.

The adipocyte hormone leptin is a critical modulator of both acute appetitive state and long-term metabolic health. Leptin was initially described as an adipostatic signal controlling food intake and energy expenditure [14]. Our previous data showed that prenatal glucocorticoid overexposure can result in liver steatosis [15]. The signal transducers and activators of transcription (STAT) pathway is thought to be the main transmission of leptin signaling [16]. Some reported that the leptin signal is terminated by induction of a family of proteins, suppressor of cytokine signaling (SOCS) through inhibiting the STAT signaling cascade [17]. The mechanisms of prenatal DEX treatment and postnatal high fat diet in the leptin, STAT and SOCS were unknown.

In this study, we examined the mechanism of postnatal high-fat diet with prenatal glucocorticoid induced liver steatosis. We investigated whether the second hit of high fat diet in the usual environment will exacerbate the liver injury. These results provide an environmental risk factor that is modifiable and that might ameliorate disease progression in nonalcoholic fatty liver disease (NAFLD) patients [18,19].

## 2. Results and Discussion

### 2.1. Higher AST and Cholesterol in DEX Treated Group and DHF Treated Group

Weight increased more in the DHF treated group (DEX plus high fat-diet) than in the DEX treated group (Table 1). The total cholesterol level increased the most in the DEX treated group and increased in the DHF treated group compared to the vehicle treated group. Triglyceride increased in the DEX treated group but did not show significant change in the DHF treated group compared to the vehicle treated group. HDL decreased in the DEX treated group and DHF treated group compared to the vehicle treated group (Table 1).

### 2.2. Liver Steatosis Was More Severe in the DHF Treated Group

**Figure 1:**
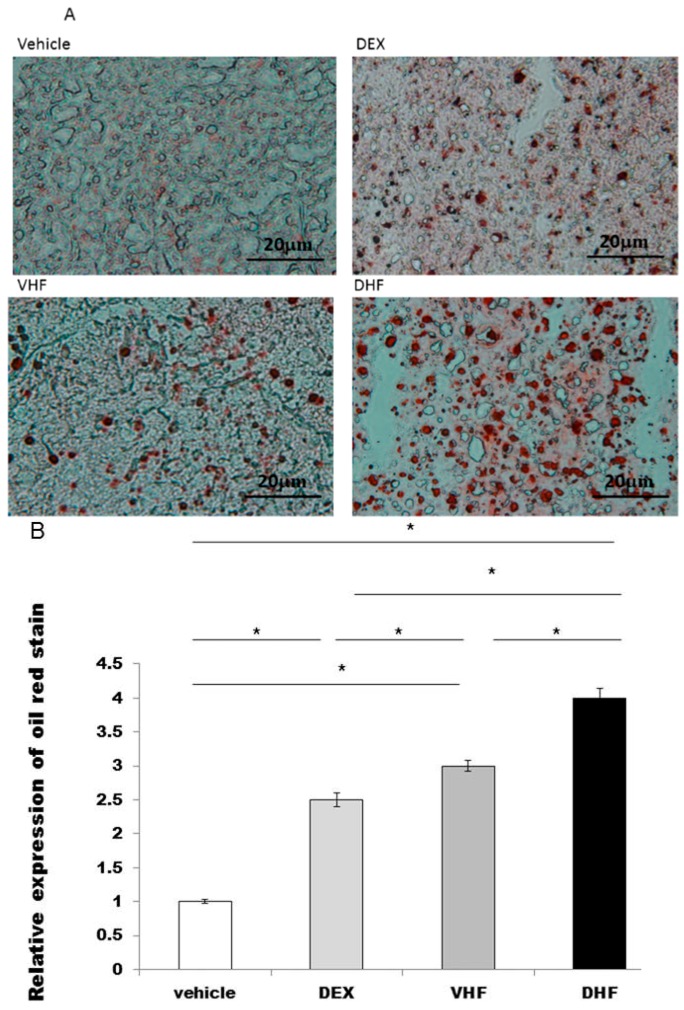
The liver steatosis was studied by oil red. (**A**) It was overexpressed in the prenatal dexamethasone (DEX) exposure plus postnatal high-fat diet group (DHF treated group), prenatal DEX exposure group (DEX treated group) and postnatal high-fat diet group (VHF treated group) than the vehicle treated group at ~180 day old rats; (**B**) Semi-quantification of the oil red stained cells. All the results represent mean ± standard error of six animals, * *p* < 0.05. The letters above each represented different groups with DEX representing the prenatal steroid treated; VHF treated group, postnatal HF diet group; DHF treated group, prenatal DEX exposure plus postnatal HF diet group (original magnification 400×; bar = 20 μm).

Liver steatosis in ~180 day old rats was studied by oil red and it was overexpressed in the DHF treated group, DEX treated group and VHF treated group (high-fat diet only) than the vehicle treated group (Figure 1A,B). More severe steatosis was found in the DHF treated group than in the DEX treated group and VHF treated group.

### 2.3. Apoptosis Was Higher in the DHF Treated Group

**Figure 2:**
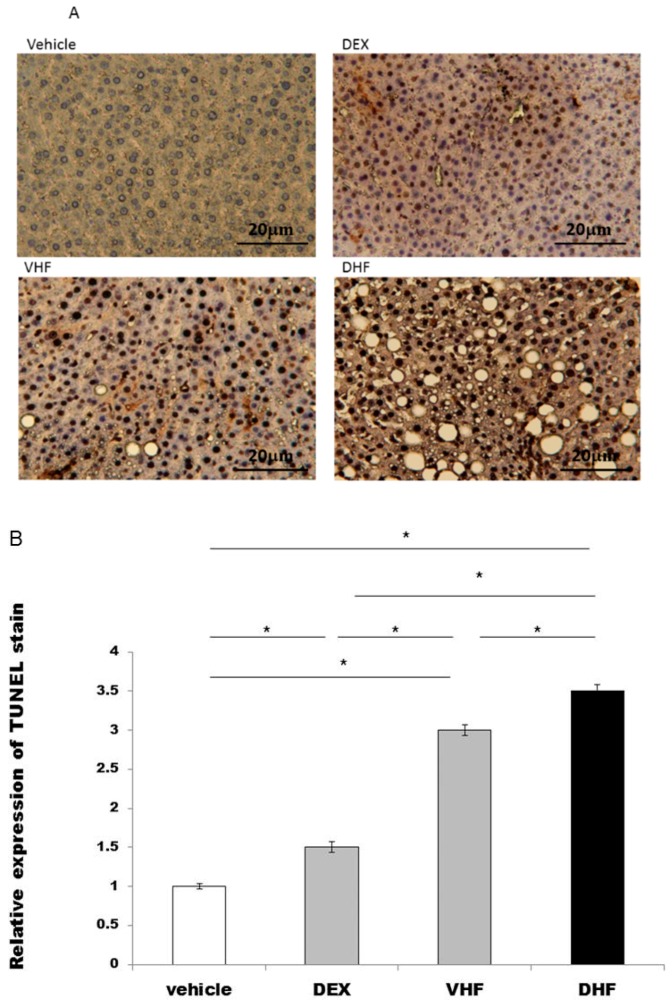
The extent of TdT-mediated dUTP Biotin Nick End Labeling (TUNEL) staining was assessed for whether apoptosis was involved in this liver damage. (**A**) It was overexpressed in the prenatal DEX exposure plus postnatal HF diet group (DHF treated group), prenatal DEX exposure group (DEX treated group) and postnatal HF diet group (VHF treated group) compared to the vehicle treated group; (**B**) Semi-quantification of the TUNEL stained cells. All the results represent mean ± standard error of six animals, * *p* < 0.05. The letters above each represented different groups as in Figure 1 (original magnification 400×; bar = 20 μm).

**Figure 3:**
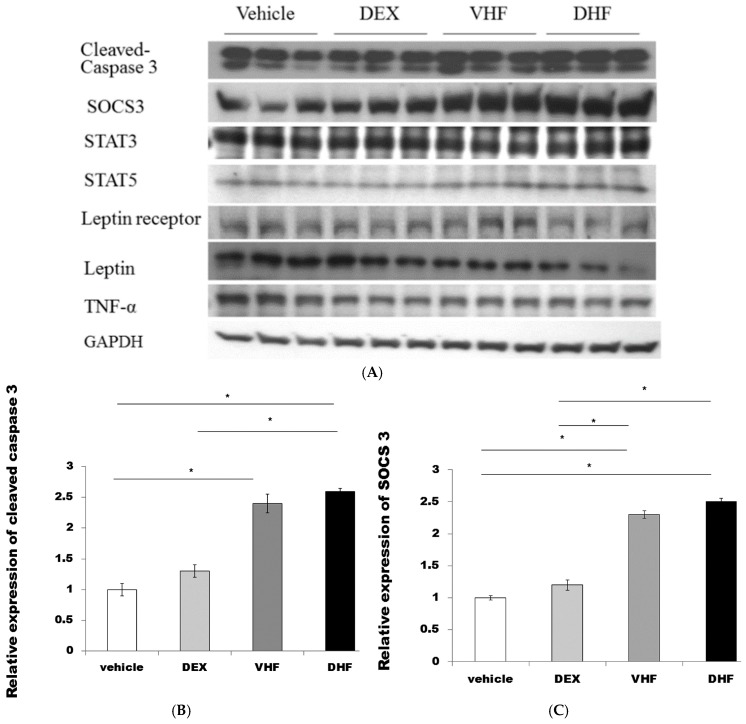

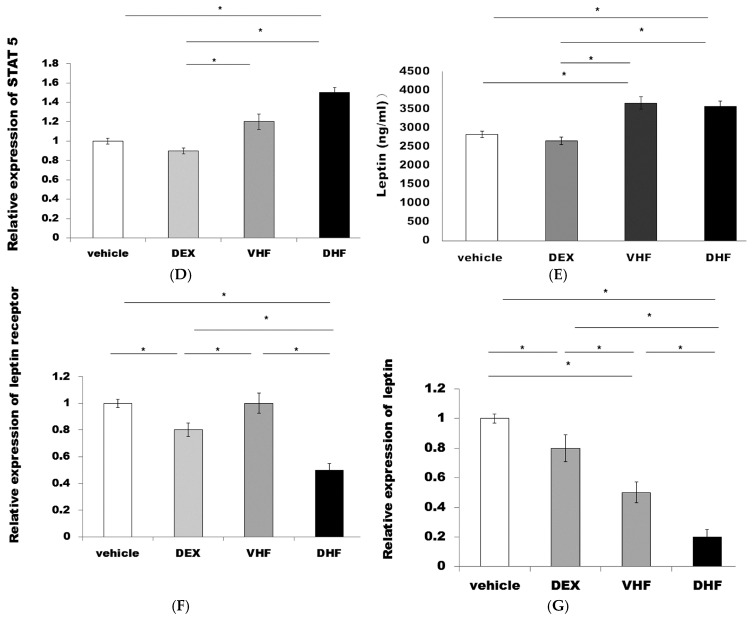
(**A**,**B**,**C**,**D**) Higher activated caspase 3, SOCS3, STAT5 expression and (**E**) serum leptin level were found in the DHF treated group and VHF treated group than in the DEX treated group and vehicle treated group. (**F**,**G**) Decreased liver leptin and leptin receptor expression was found in the prenatal DEX exposure plus postnatal HF diet group (DHF treated group) compared to the prenatal DEX exposure group (DEX treated group) and vehicle treated group. All the results represented mean ± standard error of six animals, * *p* < 0.05. The letters above each represented different groups with DEX representing the prenatal steroid treated; VHF treated group, postnatal HF diet group; DHF treated group, prenatal DEX exposure plus postnatal HF diet group.

We studied whether apoptosis was involved in this prenatal DEX exposure plus postnatal HF diet group liver damage [15]. The activation of the apoptotic machinery was measured by using the cleaved caspase 3 and the extent of TdT-mediated dUTP Biotin Nick End Labeling (TUNEL) staining. Our data showed that apoptosis was greater in the DHF treated group, DEX treated group and VHF treated group than the vehicle treated group (Figure 2A,B and Figure 3B). It was highest in the DHF treated group than in VHF treated group, DEX treated group and vehicle treated group.

### 2.4. The Higher Activated Caspase 3 (Apoptosis) and Leptin Resistance in the DHF Treated Group

Western blot showed increased SOCS3and STAT5 protein expressions were seen in the DHF treated group than in the vehicle treated group, and DEX treated group (Figure 3A,C,D). Leptin resistance was reported with high triglyceride level with lower leptin receptor [20]. This was found in our DHF treated group which had higher plasma leptin (Figure 3E) with lower liver leptin receptor level than the DEX treated group, VHF treated group and vehicle treated group (Figure 3A,F). However, there was decreased liver leptin expression in the DHF treated group, the DEX treated group and VHF treated group compared to the vehicle treated group (Figure 3A,G). There was no significant difference of TNFα and STAT3 among the four groups (Figure 3A).

### 2.5. Increased Oxidative Stress Studies in DHF Treated Group

**Figure 4:**
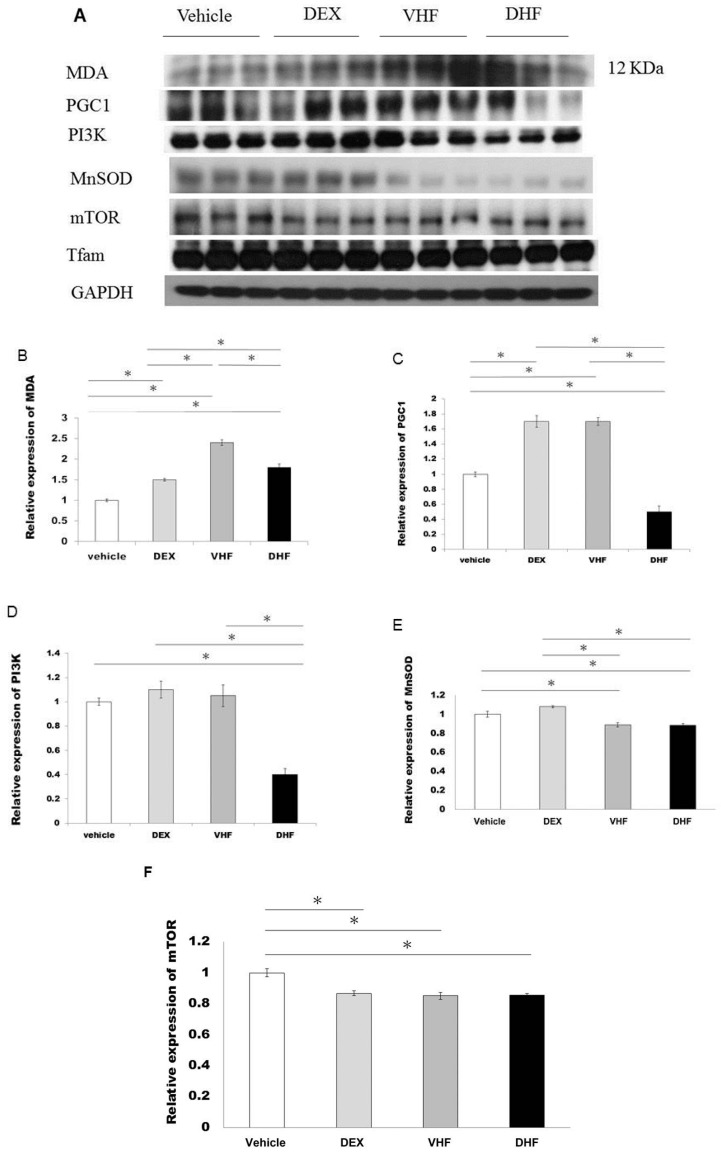
(**A**,**B**,**C**,**D**) Increased malondialdehyde (MDA) protein expression and decreased PGC1, PI3K expression were seen in the DHF treated group than in vehicle treated group, VHF treated group and DEX treated group. (**E**) Lower antioxidant MnSOD was found in the DHF treated group than in the vehicle treated group and DEX treated group. (**F**) There was no significant difference of mTOR among the four groups. All the results represent mean ± standard error of six animals, * *p* < 0.05. The letters above each represented different groups with DEX representing the prenatal steroid treated; VHF treated group, postnatal HF diet group; DHF treated group, prenatal DEX exposure plus postnatal HF diet group.

Increased lipid peroxidation (malondialdehyde, MDA) protein expression and decreased mitochondrial biogenesis PGC1, PI3K expression were seen in DHF treated group than in vehicle treated group, VHF treated group and DEX treated group (Figure 4A–D). Lower antioxidant level such as MnSOD was found in VHF treated group and DHF treated group compared to the vehicle treated group and DEX treated group (Figure 4E). There was no significant difference of mTOR (Figure 4A,F) and mitochondrial transcription factor A (Tfam) (Figure 4A) among the four groups.

### 2.6. Discussion

In our study, we found that the DHF treated group exacerbated the liver steatosis compared to the prenatal DEX treated group, and the DEX treated group increased liver steatosis compared to the vehicle treated group. It was also found that there was higher liver cell apoptosis in the DHF treated group than in the DEX treated group, and the DEX treated group had higher liver apoptosis expression than vehicle treated group. We found higher oxidative stress with MDA and lower levels of the anti-oxidative marker MnSOD in the DHF treated group than in the DEX treated group, though the DEX treated group had higher MDA without changing MnSOD levels.

It was reported that prenatal dexamethasone programs increase hepatic lipid accumulation on a high-fat diet [3], and increased the potential to develop an array of metabolic disturbances in adulthood [21]. This is also found in our study that the DHF treated group had more hepatic lipid accumulation than the high fat diet only or with prenatal steroid given. This means that a high fat diet environment with prenatal stress will lead to a more severe liver fat accumulation and injury. Several studies have reported that expression of individual gene products is different in male and female fetuses [22]. The predisposition to metabolic disease differs between the sexes, and in mice fed a very-high-fat diet the female displays more striking changes in gene expression than the male [23], and obesity [24]. We studied only males for the more reliable steatosis and obesity effects among the different groups compared to the more striking effects in female.

Hepatocellular apoptosis plays a pivotal role in the pathogenesis of NAFLD [25], and prenatal glucocorticoid induces programming of liver steatosis [15]. Our study investigated whether the addition of high fat diet to prenatal steroids further resulted in an increase in the incidence of hepatocellular apoptosis and was associated with activation of caspase 3 and TUNEL stain.

Some studies reported that offspring of dexamethasone-treated dams exhibited significant hyperleptinaemia [26,27] and that plasma leptin declined after maternal dexamethasone treatment [15,28]. Leptin deficiency in mice and humans cause morbid obesity with fatty liver, and replacement leads to a decreased food intake and increased energy expenditure [29]. In this study, we found that both leptin and leptin receptor expression decreased in the DHF treated group compared to the high fat diet and prenatal steroids given. This indicated that leptin is an important factor in the DHF treated group and may be associated with liver steatosis and cellular apoptosis.

SOCS is an inhibitor of leptin signaling [17]. Long-term fasting is associated with upregulation of leptin and SOCS expression in the liver [30]. However, increased expression of SOCS in white adipose tissues of rats with acquired obesity could have blocked leptin's lipopenic action in the leptin-resistant white adipose tissues population [31]. There was no known abnormality in the leptin receptor of rats with diet induced obesity, and in this situation, any resistance to leptin action is presumed to be at a postreceptor level [31]. An increase in SOCS in the obese mice induces SOCS-mediated resistance to certain of its extrahypothalamic actions [31]. SOCS has been shown to block the action of leptin through a principal pathway of leptin signal transduction, STAT phosphorylation [17]. SOCS is also a potential mediator of leptin resistance in obesity [32]. We found the leptin resistance with lower leptin receptor expression in the DHF treated group. In our study, leptin decreased with the increased SOCS and STAT-5 in the DHF treated group which was associated with the role of the suppression of leptin possibly via STAT-5 and SOCS.

Hepatic MDA increased in a high-fat diet with NAFLD [33,34]. Some reported that superoxide dismutase (SOD) and MDA levels were increased in NAFLD rats and decreased PI3K with oxidative stress in high-fat fed rats [35,36]. PGC1-α is also involved in mitochondrial biogenesis that is vital for cell survival [37]. In our study, we found that the DHF treated group can increase higher oxidative stress with higher MDA, decreased PGC1-α and MnSOD but not in the DEX treated group. The DHF treated group may correlate with the decreasing PI3K in the pathogenesis of oxidative stress. Tfam, in the correction of the initiation of transcription from mammalian mtDNA promoters, is necessary [38]. A previous report showed that there was no significant changes observed in the expression of Tfam in high-fat diet [39]. It remained to be proven whether Tfam acts as a transcription factor *in vivo*, especially in the liver [40]. We did not see the difference of Tfam expression levels among our study groups; this may need further study to clarify.

## 3. Experimental Section

### 3.1. Animals

This study was carried out in strict accordance with the Guide for the Care and Use of Laboratory Animals of the National Institutes of Health. The care and use of the laboratory animals strictly followed the protocol of Institutional Animal Care and Use Committee, which was approved by the animal ethics committee of the Chang Gung University, Kaohsiung, Taiwan (number 2014032403). Sprague-Dawley (SD) rats (12–16 weeks old) from BioLASCO Taiwan Co., Ltd., Taipei, Taiwan were housed and maintained in a facility accredited by the Association for Assessment and Accreditation of Laboratory Animal Care International. Virgin SD female rats with male rats were allowed to mate for 24 h. The female rats were then separated and housed individually in a standard plastic home cage. After confirmation of pregnancy, pregnant female rats were randomly assigned to two groups: vehicle treated group and dexamethasone exposure group. For dexamethasone exposure, we started intraperitoneal dexamethasone daily (0.1 mg/kg/day) at gestational day 14–21. Comparatively, the vehicle treated group group was intraperitoneal injected of normal saline daily during gestational age 14–20. The male offspring were then divided into four groups (*N* = 6, per group): the vehicle treated group, prenatal dexamethasone exposure group (DEX treated group), postnatal high fat diet group (VHF treated group), and prenatal dexamethasone exposure with postnatal high fat diet group (DHF treated group), according to whether prenatal dexamethasone and/or post-natal high fat diet exposure. Male offspring rats in VHF treated group and DHF treated group received high-fat diet (58% high-fat diet, Research Diet, New Brunswick, NJ,USA, Country, D12331) from weaning to 6 months of age as indicated. Vehicle treated and DEX treated groups received control diet (protein 23.5%, fat 4.5%, crude fiber 5.0%, crude ash 7.0%, and water 13%, Fwusow Taiwan Co., Ltd., Taichung, Taiwan).

### 3.2. Experimental Procedures and Specimen Collection

All the vehicle treated group, DEX treated group, VHF treated group and DHF treated group were sacrificed at postnatal day 180 after birth. The rats were sacrificed with ketamine to minimize suffering of the animals when infection signs or respiratory or gastrointestinal symptoms appeared. The rats were anesthetized with an intramuscular injection of ketamine (10 mg/kg; Pfizer, Hsinchu, Taiwan) for termination. Immediately after termination of the rats, the liver tissues were taken and weighted together with intercardiac blood collections and placed into EDTA-containing vials. The blood assay of triglyceride, cholesterol, high-density lipoprotein (HDL) levels and aspartate transaminase (AST) activities were determined by a standard autoanalyzer (Hitachi model 7450, Tokyo, Japan).

### 3.3. Localization of Oil Red Stain Targets Fat Deposits and Analysis

In the liver lipid proteins expression study, we cut 2~3 μm thick sections of the frozen liver tissue and mounted it on coating slides. Tissue sections were incubated with 3% hydrogen peroxide for 10 min to block endogeneous peroxidase activity. The sections were stained with Oil Red O [15]. The positive stained cell numbers were counted in a total of five hundred hepatocytes in each group.

### 3.4. TdT-Mediated dUTP Biotin Nick End Labeling (TUNEL)

The liver cellular apoptosis expression was studied. The detection of the TUNEL expression was according to the method of the protocol [15,41]. We used Apoptosis Detection Kit (CHEMICON International, Inc., Temecula, CA, USA) for TUNEL staining. Then, we washed the deparaffinized sections with distilled water and treated the slides with Protein Digestion Enzyme for 15 min at 37 °C. The positive stained cells numbers were counted in a total five hundred hepatocytes in each rat.

### 3.5. Western Blotting Analysis

Tissues were dissected from samples and frozen immediately in liquid N_2_. When used, the tissue was homogenized in a buffer and centrifuged at 14,000× *g*. Proteins (40 µg) from each samples’ supernatant were separated by SDS-PAGE. They were transferred to polyvinylidene difluoride membranes by electrophoresis. The membranes were blocked in TBST (Tris-buffered saline, 0.1% Tween 20) buffer which contained 5% low fat milk powder and this blocking process was for 1 hour at room temperature. After this, the immunoblotting assay was performed by using specific primary antibodies. The primary monoclonal mouse included cleaved caspase 3 (Cell Signaling#9661, Danvers, MA, USA), leptin (abcam/ab16227, Cambridge, UK), leptin receptor ((abcam/ab5593), TNFα (Cell Signaling#3707), STAT 3, STAT5 (abcam/ab76315, 68465), SOCS (abcam/ab16030), phosphoinositide-3-kinase (PI3K) (abcam/ab40755), MDA (abcam/ab27642), PGC1 (abcam/ab54481), Mitochondrial transcription factor A (Tfam) (Santa cruz/sc23588, Paso Robles, CA, USA) antibodies were studied. A secondary alkaline phosphatase-conjugated anti-IgG antibody (1:5000; Promega, Madison, WI, USA) followed [15,41,42]. The the Blot AP System (Promega) was used for Western blots visualization.

### 3.6. Cytokine Secretion with Enzyme-Linked Immunosorbent Assay (ELISA)

The plasma was analyzed for the levels of cytokine using leptin commercial ELISA kits (R & D Systems, Minneapolis, MN, USA), according to the manufacturer’s protocols. Each assay utilized a standard curve for using recombinant cytokine generation calculation.

### 3.7. Statistics

Data were expressed as mean ± standard error of the mean. For most parameters, statistical analysis was done using ANOVA with Bonferroni *post hoc* test for multiple comparisons depend on two or four groups were analyzed. Results with a *p*-value of less than 0.05 were considered to be statistically significant. All of the statistical tests were performed by using SPSS 15.0 for Windows XP (SPSS, Inc., Chicago, IL, USA).

## 4. Conclusions

Our study shows that postnatal high-fat diet increases liver steatosis and apoptosis threatened by prenatal dexamethasone through the oxidative effect more than with only prenatal dexamethasone. This indicates that high fat diet is an environmental risk factor for disease progression in patients with nonalcoholic fatty liver disease after prenatal stress.

## Figures and Tables

**Table 1:** Demographic features and biochemical data of the studied groups.

	Vehicle	DEX	VHF	DHF
Weight (gm)	581.6 ± 29.2 *	610.3 ± 21.6 ^†^	679.0 ± 30.7 *^,^^†^	686.1 ± 29.9 *^,†^
AST/GOT (U/L)	81.8 ± 30.5 *	107.4 ± 10.4 ^†^	146.3 ± 11.3 *^,†^	313.1 ± 43.3 *^,†^
Cholesterol (mg/dL)	78.8 ± 6.1 *	137.0 ± 4.8 ^†^	81.2 ± 7.3 *	98.0 ± 2.3 *^,†^
Triglyceride (mg/dL)	105.2 ± 19.0 *	137.8 ± 31.9 ^†^	93.3 ± 13.6 *	103.1 ± 12.5 *
HDL (mg/dL)	48.8 ± 3.6 *	38.2 ± 2.9 ^†^	48.3 ± 4.1 *	41.9 ± 2.9 ^†^

AST: aspartate transaminase, GOT: glutamate oxaloacetate transaminase, HDL: High-density lipoprotein, * *p* < 0.05 compared to DEX treated group; ^†^ *p* < 0.05 compared to vehicle treated group, mean ± standard error.
